# First evidence showing that *Pepper vein yellows virus* P4 protein is a movement protein

**DOI:** 10.1186/s12866-020-01758-y

**Published:** 2020-03-30

**Authors:** Sangsang Li, Xianyan Su, Xiangwen Luo, Yu Zhang, Deyong Zhang, Jiao Du, Zhanhong Zhang, Xian OuYang, Songbai Zhang, Yong Liu

**Affiliations:** 1 Longping Branch, Hunan University, Changsha, 410125 China; 2 Plant Protection Institute of Anhui Academy of Agricultural Science, Hefei, 230001 China; 3 Key Laboratory of Pest Management of Horticultural Crop of Hunan Province, Hunan Plant Protection Institute, Hunan Academy of Agricultural Science, No 726 Second Yuanda Road, Furong District, Changsha, 410125 Hunan province P. R. China

**Keywords:** *Pepper vein yellows virus*, P4 protein, Plasmodesmata, Movement protein, Cell-to-cell movement

## Abstract

**Background:**

Plant viruses move through plasmodesmata (PD) to infect new cells. To overcome the PD barrier, plant viruses have developed specific protein(s) to guide their genomic RNAs or DNAs to path through the PD.

**Results:**

In the present study, we analyzed the function of *Pepper vein yellows virus* P4 protein. Our bioinformatic analysis using five commonly used algorithms showed that the P4 protein contains an transmembrane domain, encompassing the amino acid residue 117–138. The subcellular localization of P4 protein was found to target PD and form small punctates near walls. The P4 deletion mutant or the substitution mutant constructed by overlap PCR lost their function to produce punctates near the walls inside the fluorescent loci. The P4-YFP fusion was found to move from cell to cell in infiltrated leaves, and P4 could complement *Cucumber mosaic virus* movement protein deficiency mutant to move between cells.

**Conclusion:**

Taking together, we consider that the P4 protein is a movement protein of *Pepper vein yellows virus.*

## Background

Plasmodesmata-mediated macromolecular trafficking is critical for plant growth and development [1]. Plant viruses have been shown to traffic between host cells through plasmodesmata (PD), and such trafficking is crucial for viral systemic infection [2].

PD is considered as a bottleneck for plant virus infection in plant, due mainly to its size exclusion limit (SEL) and/or the intricate and dynamic regulations controlled by the host defense mechanism [3]. To overcome this bottleneck, plant viruses have evolved to encode movement protein(s) (MP) to facilitate their intracellular trafficking, in a form of viral replication complexes or viral particle [4]. The MPs produced by plant viruses are strikingly different [5]. The pioneer report of viral MP is the 30 kDa MP of *Tobacco mosaic virus* (TMV), this MP was considered to guide TMV virion to move between cells [6], and its domain of 19 amino acids (195 to 213) is essential for localization of the MP to the cell wall fraction of plant cells [7]. Similarly as TMV, the Ourmiaviruses also encoded a 30 K movement protein to guide virus movement in plant cells [8]. The second type of MPs have two or three specialized MPs, and are referred to as double or triple gene block proteins (DGBps and TGBps) [9], which form polyprotein to localize to the periphery of the plant cells [10]. The third type of MPs are low molecule MPs, such as NSm encoded by *Tomato spotted wilt tospovirus* (TSWV) [11]. The transmembrane dispositon of low molecule movement protein of *Prunus necrotic ringspot virus* (PNRSV) is essential for its membrane localization, however, is not indispensable for virus cell-to-cell movement [12].

*Pepper vein yellows virus* (PeVYV), a new member in the genus *Polerovirus*, family *Luteoviridae*, was reported to infect multiple important solanaceous crops in Europe [13], Asia [14] and Africa [15]. Although the P4 protein of PeVYV was recently reported as a viral movement protein, based on the bioinformatic comparison to the genome structure of the movement protein of the type *Potato leafroll virus* species (PLRV) [16], the biological function and the key domain of P4 protein needed for PD traffiking remained unknown.

In the present study, we intend to determine the key domain needed for the interaction between the P4 protein and PD, and the capacity of P4 protein complementing *Cucumber mosaic virus* movement protein deficiency mutant to move between cells.

## Results

### P4 protein construction

Membrane association and plasmodesmata targeting are crucial functions of viral movement proteins. To investigate domain(s) in the P4 protein that are responsible for membrane association, we first predicted transmembrane (TM) domain(s) in the P4 protein using several bioinformatics algorithms. Results shown in Fig. S1 revealed two potential TM domains in the P4 protein. Both DAS and TMpred algorithms showed that the P4 protein was an integral membrane protein and the two predicted TM domains were located at a region encompassing aa residue 127–129 (by DAS algorithm) and aa residue 117–138 (by TMpred algorithm), respectively.

### P4 protein targets PD of host cells

**Fig. 1:**
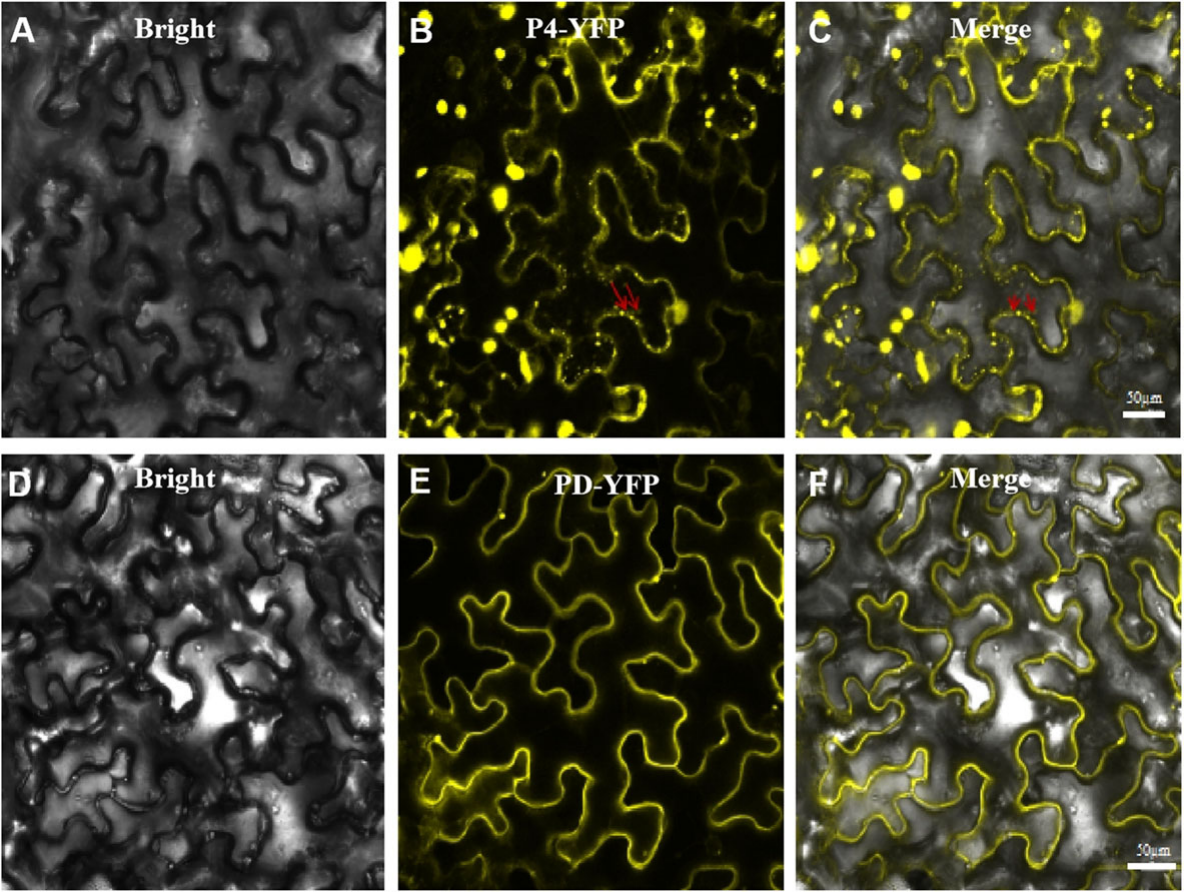
Subcellular localization patterns of P4-YFP and PD-YFP. *Nicotiana benthamiana* leaves were infiltrated with *Agrobacterium tumefaciens* GV3101 cultures carrying pP4-YFP or pPDLP8-YFP. **a**-**c**: P4-YFP subcellular localization; **d**-**f**: PD-YFP subcellular localization. Bars, 50 μm. Red arrows indicate the small punctures near the cell walls

To determine whether the P4 protein could target plasmodesmata in cell walls, we first infiltrated *N. benthamiana* leaves with *A. tumefaciens* cultures carrying the pP4-YFP or pPDLP8-YFP plasmid. The infiltrated leaves were harvested at 48 hpi and then examined for the intracellular localization patterns of the two fusion proteins by Confocal Microscopy. The result showed that the P4-YFP fusion protein accumulated as yellow fluorescence punctates in the cytoplasm and near the cell walls (Fig. 1, upper panel, red arrows showed). The PD-YFP fusion protein was found to produce small yellow fluorescence punctates near the cell walls, but not large punctates in the cytoplasm like the P4-YFP (Fig. 1, lower panel).

**Fig. 2:**
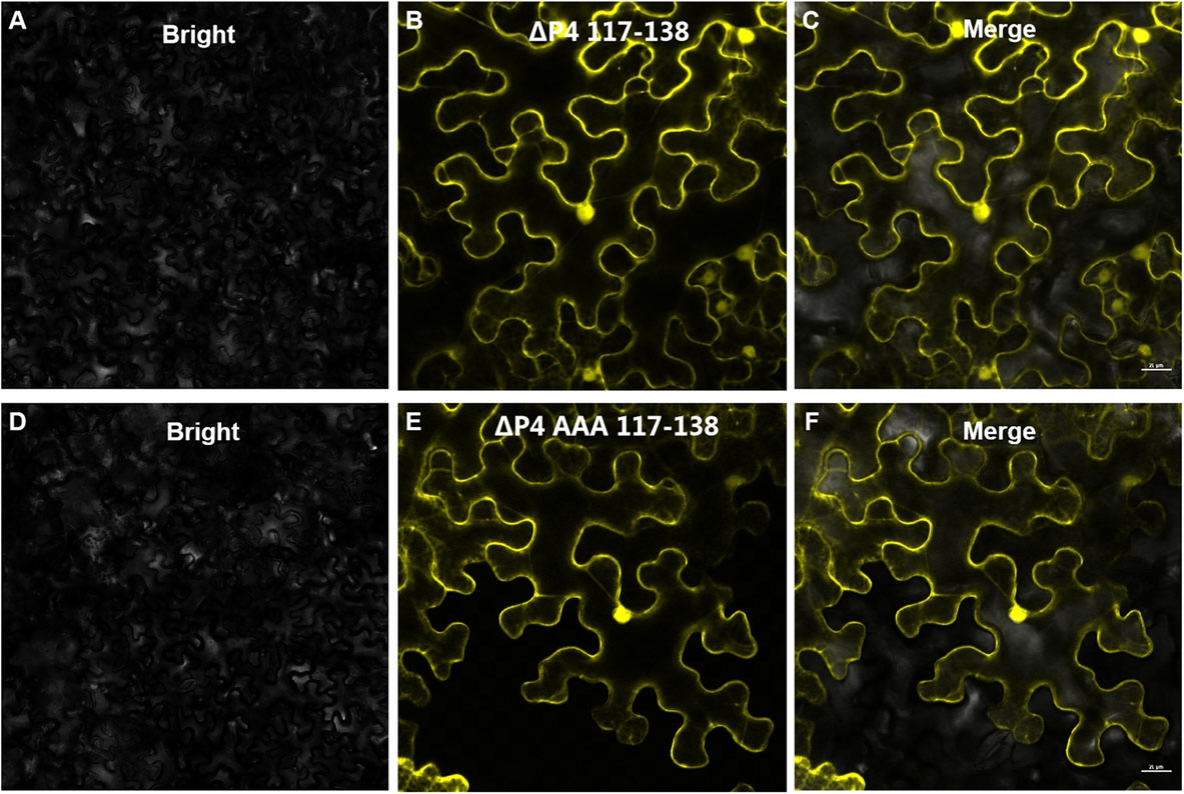
Subcellular localization patterns of ΔP4^117–138^-YFP and ΔP4^AAA117–138^-YFP. *N. benthamiana* leaves were infiltrated with *Agrobacterium tumefaciens* GV3101 cultures carrying pΔP4^117–138^-YFP or pΔP4^AAA117–138^-YFP. **a**-**c**: ΔP4^117–138^-YFP subcellular localization; **d**-**e**: ΔP4^AAA117–138^-YFP subcellular localization. Bars, 50 μm

**Fig. 3:**
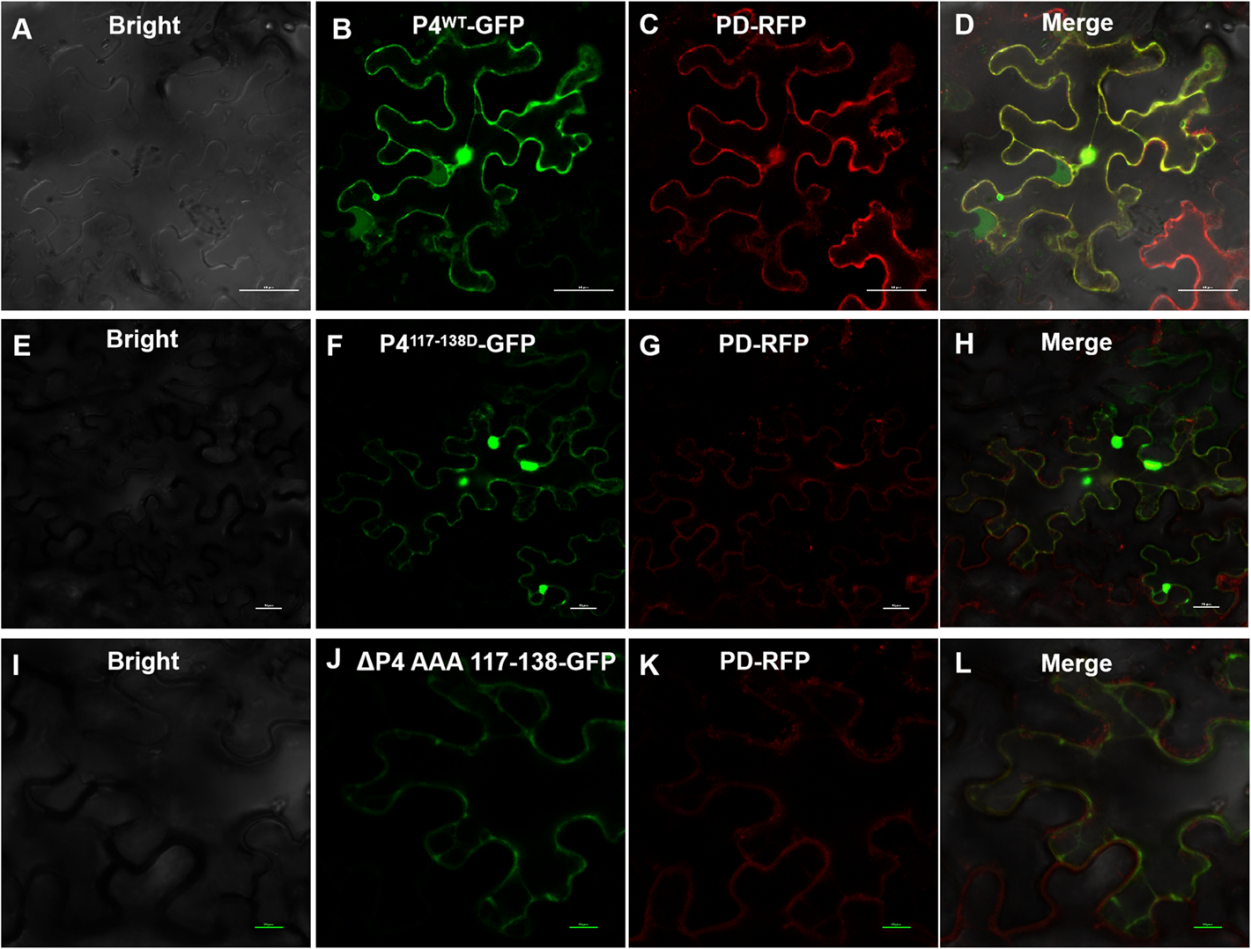
Co-localization patterns of P4, ΔP4^117–138^ or ΔP4^AAA117–138^ with PDLP8. *Nicotiana benthamiana* leaves were infiltrated with *Agrobacterium tumefaciens* GV3101 cultures carrying pΔP4^117–138^-GFP, pΔP4^AAA117–138^-GFP and pPDLP8-RFP (PD-RFP). **a**-**d**: P4-GFP colocalizating with PD-RFP (PDLP8); **e**-**h**: ΔP4^117–138^-GFP colocalizating with PD-RFP (PDLP8); **i**-**l**: ΔP4^AAA117–138^-GFP colocalizating with PD-RFP (PDLP8). Bars, 10 μm

To confirm if the computer-predicted TM domains in the P4 protein had the ability to target plasmodesmata, we fused the P4 mutants ΔP4^117–138^ and ΔP4^AAA117–138^ to YFP, respectively, and expressed them in *N benthamiana* leaves. By 48 hpi, the infiltrated leaves were harvested and examined under the confocal microscope. The results showed that the yellow fluorescence from the ΔP4^117–138^-YFP fusion or from the ΔP4^AAA117–138^-YFP fusion was near cell walls, but no yellow punctates were observed in the cell walls, reminiscent of PD localization (Fig. 2). The co-localization of P4 or its mutants with PDLP8 (marker protein localized in PD) showed that only P4 could target PD (Fig. 3).

### P4 protein functions as movement protein

To determine whether the P4 protein can move through PD, we infiltrated *N. benthamiana* leaves with *A. tumefaciens* culture carrying the pP4-YFP plasmid. As a control, we infiltrated *N. benthamiana* leaves with a different culture carrying the pPDLP8-YFP plasmid. A total of 35 fluorescent loci expressing PD-YFP were examined under the confocal microscope at 24 hpi and the PD-YFP fusion was found in single cells only (Fig. S2 and Table S2). At the same time point, the P4-YFP fusion in 21 of the 23 fluorescent signals had trafficked through the PD and entered the neighboring cells.

**Fig. 4:**
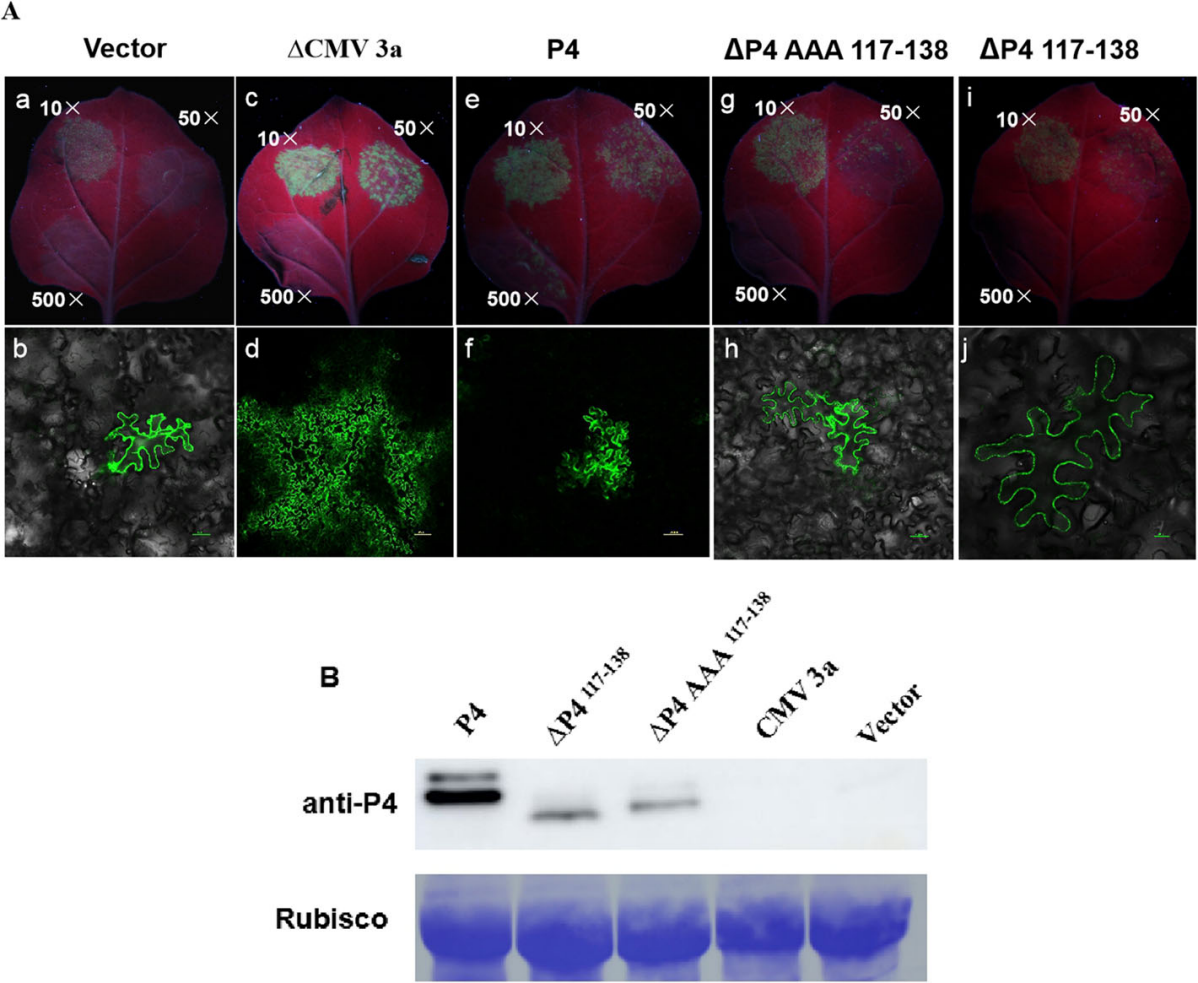
The P4 could complement movement capacity of Cucumber mosaic virus movement protein deficiency mutant between epidermal cells in *N. benthamiana* leaves. **a**: *Nicotiana benthamiana* leaves were infiltrated with *Agrobacterium tumefaciens* GV3101 cultures (10, 50 or 500 fold dilution of OD600 = 1.0); a, c, e, g, i: Complementary tests of P4 or its mutants and *Cucumber mosaic virus* movement protein deficiency mutant under UV light; b, d, f, h, j: Complementary tests of P4 or its mutants and *Cucumber mosaic virus* movement protein deficiency mutant under a confocal microscope (Bars, 10 μm or 20 μm). **b**: Western blotting detection of P4 and its mutants expressed in epidermal cells of *N. benthamiana* leaves

To further determine whether the P4 protein functions as movement protein to drive virus cell-to cell moving through PD, we infiltrated *N. benthamiana* leaves with *A. tumefaciens* culture carrying the plasmid of P4 or its mutants and CMV infectious clone with its movement protein 3a replaced by GFP. As a control, we infiltrated *N. benthamiana* leaves with a different culture carrying the plasmid of expressing P4 and its mutants. As Fig. 4 shown, P4, not P4 mutants could drive *Cucumber mosaic virus* movement protein deficiency mutant cell-to-cell moving through PD. A total of 46 of the 50 fluorescent signals expressing P4-GFP and CMV movement protein deficiency mutant were examined under the confocal microscope at 6 dpi, and the P4 mutants was found in single cells only or less than 16% virus entered the neighboring cells (Fig. 4 and Table 1).

**Table 1:** Movement of PeVYV P4 between epidermal cells in *N. benthamiana* leaves at 6 dpi

Constructs	No. of loci examined	No. of loci with a single cell (%)	No. of loci with more than 2 cells (%)	*ρ*-value
Vector	50	50^a^ (100%)	0	
CMV 3^a^	50	0	50(100%)	
P4	50	4(8%)	46(92%)	< 0.05^b^
∆P4 117–138	50	50(100%)	0	
∆P4 AAA117–138	50	42(84%)	8(16%)	< 0.05

^a^ Loci with signal cells expressing GFP fluorescence

^b^*ρ*-value was determined using the unpaired two-tailed Student *t*-test

## Discussion

It is well documented that different plant viruses encode different movement proteins (MPs) to traffic through PD between two cells [6, 9, 11]. For example, the 17 kDa movement protein of *Potato leafroll virus* (PLRV, genus *Polenovirus*, family *Luteoviridae*), was reported as a low molecular weight movement protein of PLRV [17]. In the present study, the PeVYV P4 protein (17.5 kDa), which was previously predicted as a virus movement protein, was analyzed and found to localize to PD in cell walls. A domain encompassing amino acid residues 117–138 in the P4 protein was determined to have an ability to target PDs.

The P4-YFP fusion protein was found to traffic between epidermal cells, and P4 could complement heterological *Cucumber mosaic virus* movement protein deficiency mutant to move between cells. These results provided the first evidence showing that the PeVYV 17.5 kDa protein is a viral MP.

Interestingly, in the *N. benthamiana* leaf epidermal cells expressing P4-YFP fusion protein, large and small yellow fluorescent punctates were observed (Fig. 2). Previous studies have shown that many viruses can produce aggregates during their infection in plant and these aggregates may have multiple functions including preventions of degradations by host cellular machinery [18] and/or providing viral and host factors for virus replication [19]. So the large punctates were found in cytoplasm and the small punctates were found near the cell walls.

## Conclusion

The results presented here demonstrated that the 17.5 kDa protein of PeVYV is a movement protein of the virus. Whether this protein also has a role in virus replication and/or counteraction of host defense machinery requires further investigation.

## Methods

### Plants

*Nicotiana benthamiana* plants were grown inside a culture room set at 25 °C with a 16 h light/8 h dark illumination. The seeds of *N. benthamiana*, which is a laboratory cultivar were obtained from The Plant Virology Laboratory of Ningbo University, Ningbo, China and stored in our lab. When the plants reached six-eight weeks old, they were infiltrated individually with one of the agrobacterium cultures made in this study.

### P4 protein construction prediction

Transmembrane (TM) domain inside the P4 protein was predicted using five commonly used methods available on Internet: DAS (http://www.sbc.su.se/miklos/DAS), HMMTOP (http://www.enzim.hu/hmmtop/), TMpred (https://embnet.vital-it.ch/software/TMPRED_form.html), Split (http://split.pmfst.hr/split/4), and Topcons (http://topcons.cbr.su.se).

### Plasmid constructs and agroinfiltration

Full length PeVYV *P4* gene and its alanine substitution mutant or deletion mutant aa position (117–138) were constructed individually through overlap PCR using primers listed in Table S1. The wild type (WT) P4 and its mutants were cloned into expression vector pGWB441(YFP) or pGWB505(GFP) using the Gateway technology [20]. Plasmid pPDLP8-YFP and pPDLP8-RFP (marker protein localized in PD) was from a previously described source and was used to localize plasmodesmata in cell walls [21]. These plasmids were introduced individually into *Agrobacterium tumefaciens* strain GV3101. *Agrobacterium* cultures containing the corresponding plansmid were grown overnight in a YEP medium (10 g yeast extract, 10 g Bacto peptone and 5 g NaCl in one liter H_2_O, pH 7.0) supplemented with 100 mg/L kanamycin and 50 mg/L rifampicin. *Agrobacterium* cells were pelleted and then incubated for 3 hours in an infiltration buffer (10 mM MgCl_2_, 10 mM MES, pH 5.9, and 150 μM acetosyringone). The cultures were further diluted to OD600 = 0.2 and then infiltrated individually into the abaxial side of *N. benthamiana* leaves. The infiltrated plants were again grown at 25 °C with a 16 h light/8 h dark illumination.

### Western blotting

Western blotting was operated as previously described [5]. Total protein was separated by electrophoresis in 10% SDS-PAGE and transferred onto a PVDF membrane. The antigens on the PVDF membrane were detected with polyantibody against PeVYV P4, then incubated by AP-coupled goat anti-mouse IgG (1:5000 dilution; Sigma) and 5-bromo-4-chloro-3-indolylphosphate/nitroblue tetrazolium (NBT/BCIP) staining (Sangon Biotech, Shanghai, China).

### YFP, GFP and RFP images

Agro-infiltrated leaves were detached from the plants and examined for fluorescence from different YFP, GFP or RFP fusion proteins under a Nikon TI-E + C2 confocal laser-scanning microscope (Nikon Microsystems, Watford, United Kingdom). The excitation wavelength was set at 514 nm for YFP and GFP, and 555 nm for RFP, and the emission wavelength was set at 520–550 nm for YFP and GFP, and 583 nm for RFP. The images presented in the figures are either single image or multi-layered images to achieve the maximum signal intensity. The statistics of GFP signals were determined using the unpaired two-tailed Student *t*-test.

## Supplementary information

**Additional file 1: Table S1.** Primers for constructing P4 and P4 variants expressing vector. **Table S2.** Movement of PeVYV P4 between epidermal cells in *N. benthamiana* leaves at 24 hpi. **Figure S1.** Transmembrane domains predicted in the PeVYV P4 protein by various algorisms. Positions of the predicted transmembrane domains are indicated. **Figure S2.** The P4-YFP fusion could move between epidermal cells in *N. benthamiana* leaves. *N. benthamiana* leaves were infiltrated with agrobacterium cultures carrying pP4-YFP or pPDLP8-YFP. The infiltrated leaves were harvested at 24 hpi and examined under a confocal microscope. Images showing PDLP8-YFP expression are shown in the lower panel and images showing P4-YFP expression are shown in the upper panel. Bars, 20 μm.

## Data Availability

All data generated or analyzed during this study are included in this published article and its supplementary information files.

## Abbreviations

aa: Amino acid
DGBps: Double gene block proteins
PeVYV: Pepper vein yellows virus
PD: Plasmodesmata
PLRV: *Potato leafroll virus*
TGBps: Triple gene block proteins
TM: Transmembrane
TSWV: *Tomato spotted wilt tospovirus*
