# Supplementary material for: First evidence showing that Pepper vein yellows virus P4 protein is a movement protein
Source: BMC Microbiol. 2020 Mar 30;20:72. doi: 10.1186/s12866-020-01758-y (PMC7106754; doi:10.1186/s12866-020-01758-y)

Supporting Materials

Tables

Table S1 Primers for constructing P4 and P4 variants expressing vector

Primer name	Primer sequence (5'-3')	Product
P4-attB1	GGGGACAAGTTTGTACAAAAAAGCAGGCT TCATGGAAATGGTGGATCACGTAAC	P4
P4-attB2	GGGGACCACTTTGTACAAGAAAGCTGGGT CCCCCGTTAATCTGCGAAGC	
ΔP4 117-138-FWR	GATTGGAGACTAGAGTGGGGGTCCAGCTC GTAAGCG	P4 variant with 117-138 aa deletion
ΔP4 117-138-BCF	GCTGGACCCCCACTCTAGTCTCCAATCCAC CCTGCGCA	
ΔP4 AAA117-138-FWR	GATTGGAGACTAGTGCTGCTGCAGTGGGGG TCCAGCTCGTAAGCG	P4 variant with 117-138 aa replaced by glycine
ΔP4 AAA 117-138-BCF	CTGGACCCCCACTGCAGCAGCACTAGTCTC CAATCCACCCTGCGCA	

Table S2 Movement of PeVYV P4 between epidermal cells in *N. benthamiana* leaves at 24 hpi

Constructs	No. of loci examined	No. of loci with a single cell (%)	No. of loci with more than 2 cells (%)	ρ-value
P4-YFP	23	2[a] (8.70%)	21 (91.30%)	<0.05[b]
PD-YFP	35	35 (100%)	0	

[a] Loci with signal cells expressing YFP fluorescence.

[b] ρ-value was determined using the unpaired two-tailed Student *t*-test.

Figure legends

Figure S1 Transmembrane domains predicted in the PeVYV P4 protein by various algorisms. Positions of the predicted transmembrane domains are indicated.

Figure S2 The P4-YFP fusion could move between epidermal cells in *N. benthamiana* leaves. *N. benthamiana* leaves were infiltrated with agrobacterium cultures carrying pP4-YFP or pPDLP8-YFP. The infiltrated leaves were harvested at 24 hpi and examined under a confocal microscope. Images showing PDLP8-YFP expression are shown in the lower panel and images showing P4-YFP expression are shown in the upper panel. Bars, 20 µm.

Figure S1

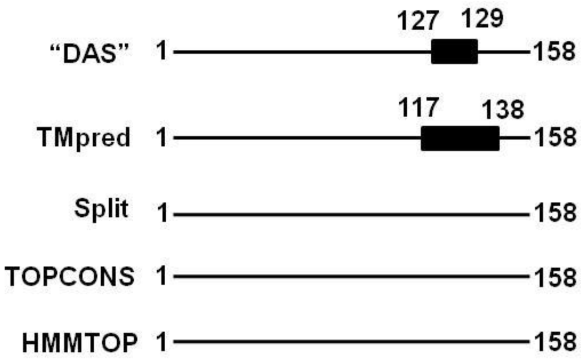

127
129
"DAS"
1
158
117
138
TMpred
1
158
Split
1
158
TOPCONS
1
158
HMMTOP
1
158

Figure S2

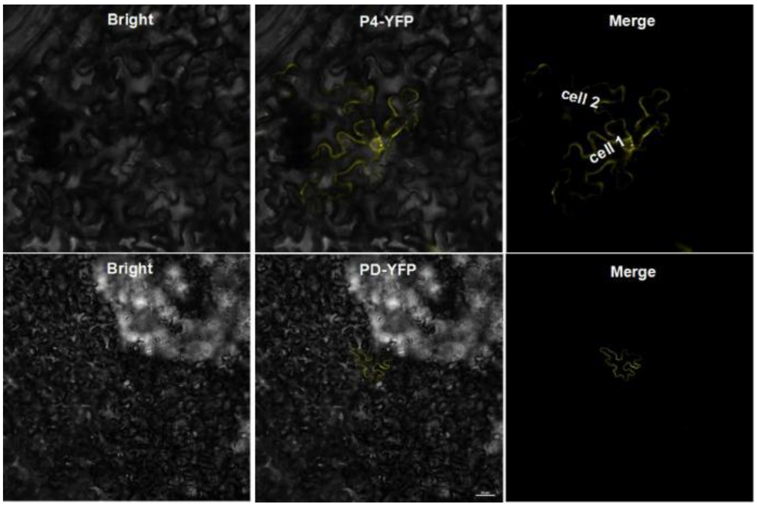

Supplement: Supplementary file 1 — Additional file 1: Table S1. Primers for constructing P4 and P4 variants expressing vector. Table S2. Movement of PeVYV P4 between epidermal cells in N. benthamiana leaves at 24 hpi. Figure S1. Transmembrane domains predicted in the PeVYV P4 protein by various algorisms. Positions of the predicted transmembrane domains are indicated. Figure S2. The P4-YFP fusion could move between epidermal cells in N. benthamiana leaves. N. benthamiana leaves were infiltrated with agrobacterium cultures carrying pP4-YFP or pPDLP8-YFP. The infiltrated leaves were harvested at 24 hpi and examined under a confocal microscope. Images showing PDLP8-YFP expression are shown in the lower panel and images showing P4-YFP expression are shown in the upper panel. Bars, 20 μm. [file 12866_2020_1758_MOESM1_ESM.pdf]
